# Development of a Novel Rabbit Model of Abdominal Aortic Aneurysm via a Combination of Periaortic Calcium Chloride and Elastase Incubation

**DOI:** 10.1371/journal.pone.0068476

**Published:** 2013-07-02

**Authors:** Yonghua Bi, Hongshan Zhong, Ke Xu, Zhen Zhang, Xun Qi, Yonghui Xia, Ling Ren

**Affiliations:** 1 Department of Radiology, The First Affiliated Hospital of China Medical University, Shenyang, China; 2 Department of Interventional Radiology, The First Affiliated Hospital, Zhengzhou University, Zhengzhou, China; 3 Key Laboratory of Diagnostic Imaging and Interventional Radiology of Liaoning Province, Shenyang, China; 4 Department of Ultrasound, The First Affiliated Hospital of China Medical University, Shenyang, China; Scuola Superiore Sant’Anna, Italy

## Abstract

**Background:**

The purpose of this study was to introduce a novel, simple and effective technique for creating a reliable rabbit model of abdominal aortic aneurysm (AAA) via a combination of periaortic calcium chloride (CaCl_2_) and elastase incubation.

**Methods:**

Forty-eight New Zealand white rabbits were divided into four groups. The AAA model was developed via a 20-minute periaortic incubation of CaCl_2_ (0.5 mol/L) and elastase (1 Unit/µL) in a 1.5-cm aortic segment (Group CE). A single incubation of CaCl_2_ (Group C) or elastase (Group E) and a sham operation group (Sham Group) were used for the controls. Diameter was measured by serial digital subtraction angiography imaging on days 5, 15 and 30. Animals were sacrificed on day 5 and day 30 for histopathological and immunohistochemical studies.

**Results:**

All animals in Group CE developed aneurysm, with an average dilation ratio of 65.3%±8.9% on day 5, 86.5%±28.7% on day 15 and 203.6%±39.1% on day 30. No aneurysm was found in Group C, and only one aneurysm was seen on day 5 in Group E. Group CE exhibited less intima-media thickness, endothelial recovery, elastin and smooth muscle cell (SMC) content, but stronger expression of matrix metalloproteinase-2, matrix metalloproteinase-9 and RAM11 compared to the controls.

**Conclusions:**

The novel rabbit model of AAA created by using a combination of periaortic CaCl_2_ and elastase incubation is simple and effective to perform and is valuable for elucidating AAA mechanisms and therapeutic interventions in experimental studies.

## Introduction

Abdominal aortic aneurysm (AAA), defined as a permanent localized aortic dilation with a diameter of 1.5 times the normal aorta diameter, is a silent degenerative disease that can be life-threatening [1]. Although human AAA is histologically characterized by adventitial inflammation, medial attenuation, elastic fiber destruction and subsequent dilation [2], the pathogenesis of AAA is not completely understood. Therefore, it is critical to develop reliable and reproducible experimental models to study AAA pathogenesis and to mimic clinical scenarios for translational research.

Several small animal models have been developed to assist in understanding the mechanisms of AAA pathogenesis. Anidjar et al. [3] first introduced an elastase-induced AAA, one of the most commonly used models in rats. This model requires insertion of a cannula and blockage of blood circulation during an extended elastase perfusion (2 hours), making the surgical procedure difficult and complex. Periarterial elastase incubation is also used to create an aneurysm in the carotid [4], [5], [6], [7] and aortic arteries [8], [9], [10], [11] in small animals. However, a lengthy elastase incubation (1–3 hours) in these models may lead to high mortality of the animals and failure of the surgical procedure. Origuchi et al. [9] also noted that periarterial elastase-induced AAA heals spontaneously.

The CaCl_2_-induced AAA model, which was first introduced by Gertz et al. [12] in the rabbit carotid artery and was subsequently used in the aortic artery [13], is another popular model. Unfortunately, periaortic CaCl_2_ application does not always induce reliable AAA formation. Isenburg et al. [14] reported that only 8 of 12 rats developed aneurysm (66.7%), even using an arbitrary threshold of a 20% diameter increase to define AAA. Tanaka et al. [15] introduced a novel rat AAA model induced by a combination of intraluminal elastase infusion and extraluminal CaCl_2_ exposure. However, this model is still complex because of the insertion of an SP10 polyethylene catheter into the femoral artery, and rabbits are more suitable than rats for investigating the pharmacologic or gene therapeutic effects of drug-eluting stents and stent graft-mediated gene delivery systems [16], [17], [18], [19]. The purpose of this study was to investigate whether a combination of periaortic CaCl_2_ and elastase incubation could work in concert to develop suitable AAA formation in rabbits.

## Materials and Methods

### Animals and Experimental Groups

Forty-eight New Zealand white rabbits (weighing 2–3 kg) from our laboratory animal center were randomly divided into four groups. Experimental models of AAA (Group CE, n = 12) were developed via a 20-minute periaortic incubation of CaCl_2_ (0.5 mol/L; Sigma-Aldrich, Shanghai, China) and elastase (1 Unit/µL; ≥30 units/mg, Shanghai Kayon Biological Technology Co., Ltd., Shanghai, China). A single 20-minute periaortic incubation of 0.5 mol/L CaCl_2_ (Group C, n = 12) or 1 Unit/µL elastase (Group E, n = 12) was used as the control. Rabbits incubated with saline solution for 20 min were used as the sham operation group (Sham Group, n = 12).There was no procedural difference among the groups except for treatment allocation. Animal care followed the Chinese Community Standard for care and use of laboratory animals, and the protocols for animal experimentation were approved by the Animal Care and Use Committee of the China Medical University. All surgery was performed under sodium pentobarbital anesthesia, and all efforts were made to minimize suffering.

### A Novel AAA Model

The rabbits were housed in individual cages at room temperature under a 12-hour dark and light cycle. Food and water were available ad libitum before and after surgery. Rabbits were anesthetized with an ear vein injection of sodium pentobarbital at a dose of 30 mg/kg. The abdominal cavity was exposed by a midline abdominal incision under sterile condition. A 1-cm aortic segment proximal to the bifurcation of the renal arteries was isolated and the lumbar arteries derived from the segment were then ligated. The isolated region of the aorta was then circumferentially wrapped with a piece of sterile gauze onto which 70 microlitres of the CaCl_2_ and/or elastase solution, or saline solution, were added depending on the group, and incubated for 20 min. Thereafter, the gauze was removed and the arterial segment was washed twice with physiological saline. The abdominal incision was closed with a continuous running suture, and the rabbits were placed in warming cages to recover.

### Intravenous Digital Subtraction Angiography

All rabbits underwent serial intravenous digital subtraction angiography imaging 5, 15 and 30 days after surgery. Rabbits were anesthetized as above (sodium pentobarbital; i.v., 30 mg/kg bolus). About 5 ml of iodinated contrast material was manually injected into the ear vein through an angiocatheter within 3–4 seconds. A 1 cm length external sizing device was placed under the abdomen, and the aortic inner diameter was measured in reference to it. Aneurysm diameter was defined as the greatest dimension of the transverse minor axis of incubated aorta, and normal aortic diameter was referrenced to the diameter of aorta just above and proximal to the aneurysm. Both diameters were measured by a person blinded to the treatment groups as previously reported [20], [21]. Successful AAA formation was defined as a focal dilation ratio of the incubated aorta with a diameter of at least 50 percent of the normal diameter.

### Animal Sacrifice and Histopathology

Five and thirty days after surgery, a second laparotomy was performed after imaging and a catheter was then introduced into the abdominal aorta at the level of the renal arteries. After euthanasia by intravenous injection of an overdose of sodium pentobarbital, pressure perfusion fixed with 10% buffered paraformaldehyde solution was processed. Aortic sections (5 µm) were stained with hematoxylin and eosin (HE) for general appearance, elastic van-Gieson (EVG) dye for elastin, and picrosirius red (PSR) for collagen under conventional light or polarized microscopy. Images of the sections were analyzed by using ImageJ 1.41 software as previously described [15]. In the HE-stained sections, the media thickness, intima thickness and intima-media thickness were measured as the average thickness of 10 points of cross-sectional aortic area. Semiquantitative analyses for elastin and collagen content were performed and calculated by measuring the elastin and collagen area in the EVG-stained and PSR-stained sections.

### Immunohistochemical Analysis

Tissue sections were deparaffinized in xylene and rehydrated through graded alcohol washes, and incubated with 1% H_2_O_2_ in methanol for 20 min to block endogenous peroxidase activity. Non-specific binding was blocked with 5% goat serum for 30 min at room temperature, and then matrix metalloproteinase-2 (MMP2), matrix metalloproteinase-9 (MMP9) (1∶200 and 1∶300 diluted in PBS, respectively; mouse monoclonal antibody; Abcam, HongKong, China), RAM11 (1∶100 diluted in PBS, code M0633, Dako) and CD31 (ready-to-use, code IR610, Dako) antibodies were incubated overnight in a humid chamber at 4°C overnight. After a wash in PBS, sections were incubated with biotinylated anti-mouse second antibody (MaiXin Bio, Fuzhou, China) for 20 min followed by the SP method according to the manufacture’s protocol. Diaminobenzidine tetrahydrochloride was used to visualize the sections and counterstaining with hematoxylin was performed, after which the sample was coverslipped. All slides were examined and scored at ×200 magnification. Any slides that exhibited strong positive immunostaining expression (>50%) were scored as 3, moderate positive expression (10%–50%) was scored as 2 and weak expression (<10%) as was scored as 1. CD31^+^ microvessels in the aortic wall were counted by light microscopy with ×200 objective. Endothelial recovery was calculated by length of CD31^+^ layer divided by the length of internal elastic lamina.

### Immunofluorescent Analysis of Smooth Muscle Cells

After blocking endogenous peroxidases, sections were incubated at 4°C overnight with mouse monoclonal anti-alpha smooth muscle actin (1∶150 diluted in PBS, clone 1A4, Sigma), followed by incubation with FITC-conjugated goat anti-mouse IgG to identify smooth muscle cells (SMCs). SMC content was calculated by measuring the alpha-actin positive area at ×200 magnification using ImageJ 1.41 software.

### Statistical Analysis

All data were expressed as means ± standard deviation. Unpaired t-tests and one-way ANOVA and two-way repeated-measures ANOVA followed by Bonferroni post-hoc tests were used for statistical analysis (Prism 5.0, GraphPad Software, Inc., SanDiego, CA). Differences were considered statistically significant at *P*<0.05.

## Results

### Inner Diameter Follow-up

**Figure 1:**
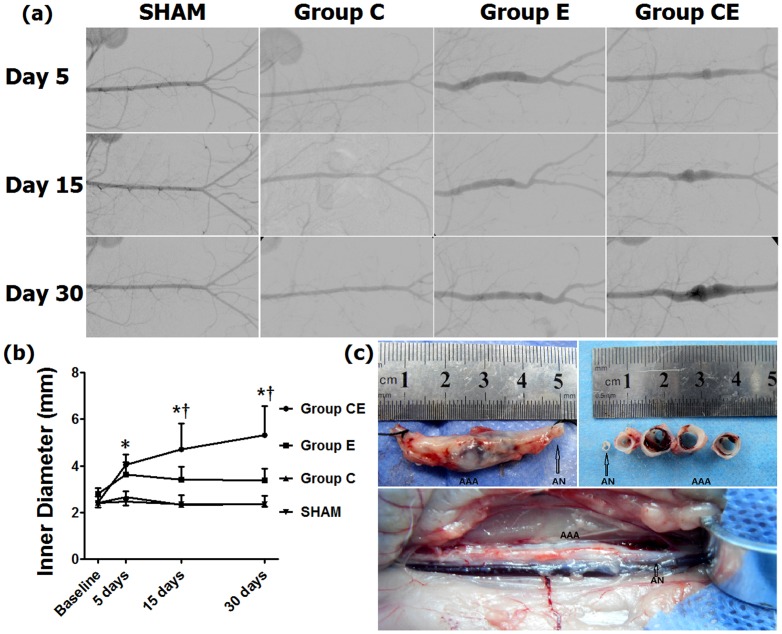
Inner diameter changes measured by intravenous digital subtraction angiography imaging. (**a**) Anteroposterior aortography shows inner diameters of incubated aorta in each group. (**b**) Profile plots of inner diameter changes in three groups. (**c**) Aneurysm sample with a diameter of 9.2 mm harvested from Group CE on day 30. AAA: abdominal aortic aneurysm; AN: aneurysm neck. *P<0.01, compared to Group C and the Sham Group, ^†^ *P*<0.05, compared to Group E. Baseline: normal aorta diameter just above and proximal to the incubated segment.

All rabbits survived the surgical procedure and no rabbits died during imaging. All animals developed AAA in Group CE by day 5, but no aneurysm was seen in Group C (Figure 1). In Group E, only one aneurysm was found on day 5, with a diameter of 4.70 mm, an increase of 58.25%, which decreased to 4.07 mm (37.04%) on day 15 and to 3.97 mm (33.67%) on day 30. The incubated segments showed gradual shrinkage in Groups C and E, however aneurysms in Group CE were found to have progressed during a 30-day follow-up. A repeated measure ANOVA revealed significant main effects of group and time, as well as significant group × time interaction (*P*<0.01). Aortic diameters in Group CE increased significantly compared to Group C and the Sham Group after day 5 (*P*<0.01) and Group E on days 15 and 30 (*P*<0.05). There were no significant differences between Group C and Group E over the 30 days (*p*>0.05).

### Aortic Wall Thickness Changes

**Figure 2:**
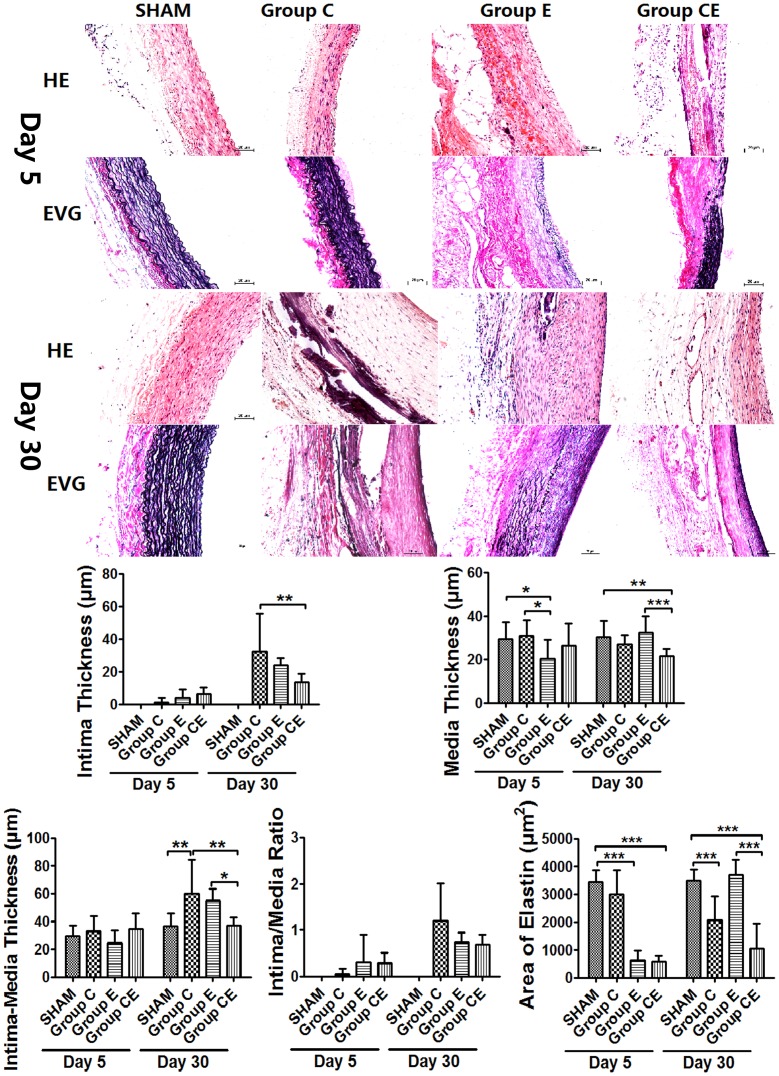
Hisopathological findings of incubated segments and their semiquantitative analysis results. HE: Hematoxylin and eosin stain, EVG: Elastic Van-Gieson stain. *P<0.05, **P<0.01, ***P<0.0001. Bar = 20 µm, original magnification ×200.

Histological examination of Group CE revealed that the aortic wall was disorganized and the number of medial SMCs was decreased on day 5. Periaortic incubation of 0.5 mol/L CaCl_2_ (Group C) caused extensive intimal thickening and medial calcification of the aortic wall but no aneurysmal dilatation. Compared to Group C (60.8±9.7 µm) and Group E (55.4±3.4 µm), the incubated segments in Group CE dilated with a significant lower intima-media thickness (36.9±2.4 µm, *P*<0.05) on day 30. Media thickness in Group E decreased significantly 5 days after surgery compared to Group C (*P*<0.05) and the Sham Group (*P*<0.05), which increased to the normal level of Sham Group on day 30. In Group CE, media thickness attenuated more significantly than that in Group E (*P*<0.0001) and the Sham Group (*P*<0.01), and hyperplastic intima thickness was thinner compared to Group C (*P*<0.01) after 30 days (Figure 2).

### Elastin Degeneration and Regeneration

EVG staining showed that elastin content decreased significantly in Group E and Group CE on day 5 compared to Group C and the Sham Group (*P*<0.0001), which increased significantly after 30 days in Group E (*P*<0.05, vs. day 5). Elastin content in Group CE was significantly less than that in Group E and the Sham Group (*P*<0.0001) on day 30. Elastin content in Group C decreased gradually after surgery, and this decrease was significant after 30 days compared to the Sham Group (*P*<0.0001). In Group E the elastic externa and lamella in media were almost nonexistent on day 5, with many disorganized newborn elastic fibers showing in the hyperplastic intima on day 30. The medial elastic lamella were fragmented in Group C, and almost gone in Group CE after 30 days, but regeneration of elastin fibers was not obvious in the hyperplastic intima.

### Change of Type I and Type III Collagen Fibers

**Figure 3:**
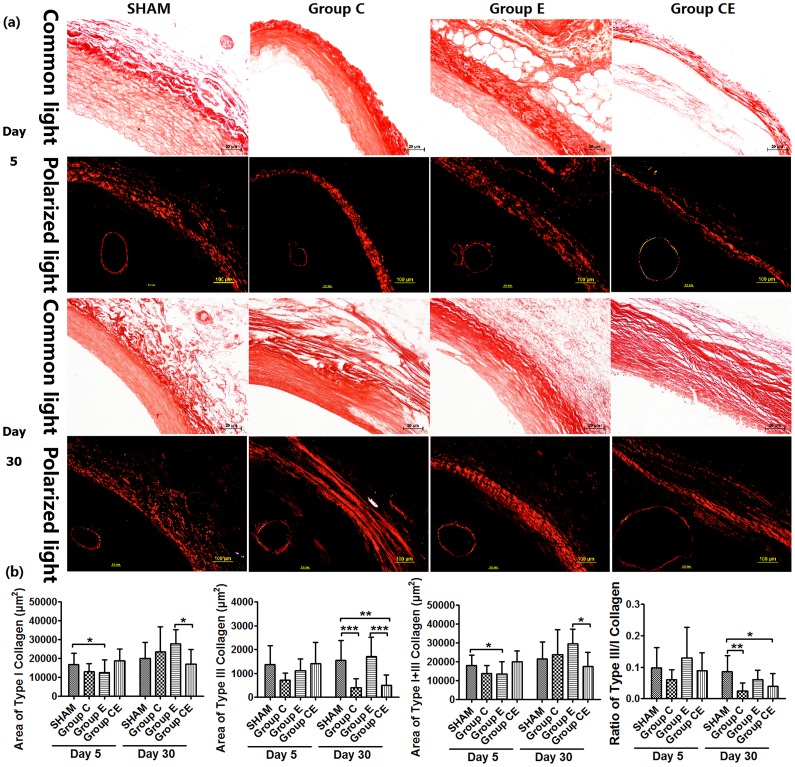
Micrographs of PSR-stained sections viewed with conventional light and circularly polarized light (a) and semiquantitative analysis of type I and type III collagen content (b). *P<0.05, **P<0.01, ***P<0.0001. All micrographs were viewed at magnification ×200, except for entire aorta ring viewed with polarized light (×20).

Collagen fibers appeared clearly and were easily distinguished in the PSR-stained sections viewed with circularly polarized light. When examined in brightfield microscopy, type I and type III collagen appeared orange-red and yellow after PSR staining (Figure 3). Type I collagen in Group E decreased on day 5 (*P*<0.05, vs. the Sham Group), and increased significantly after 30 days compared to day 5 (*P*<0.0001), and Group CE (*P*<0.05). In Group C and Group CE, type III collagen content decreased significantly on day 30 (*P*<0.0001, *P*<0.01 vs. the Sham Group, respectively), this tendency was in agreement with that calculated by using ratio of type III/I collagen (*P*<0.01, *P*<0.05 vs. the Sham Group, respectively). The changes of total collagen content were similar to the results of type III collagen content, because type III collagen accounted for less than 10% of type I collagen.

### MMP2, MMP9, CD31 Expression and Inflammatory Infiltration

**Figure 4:**
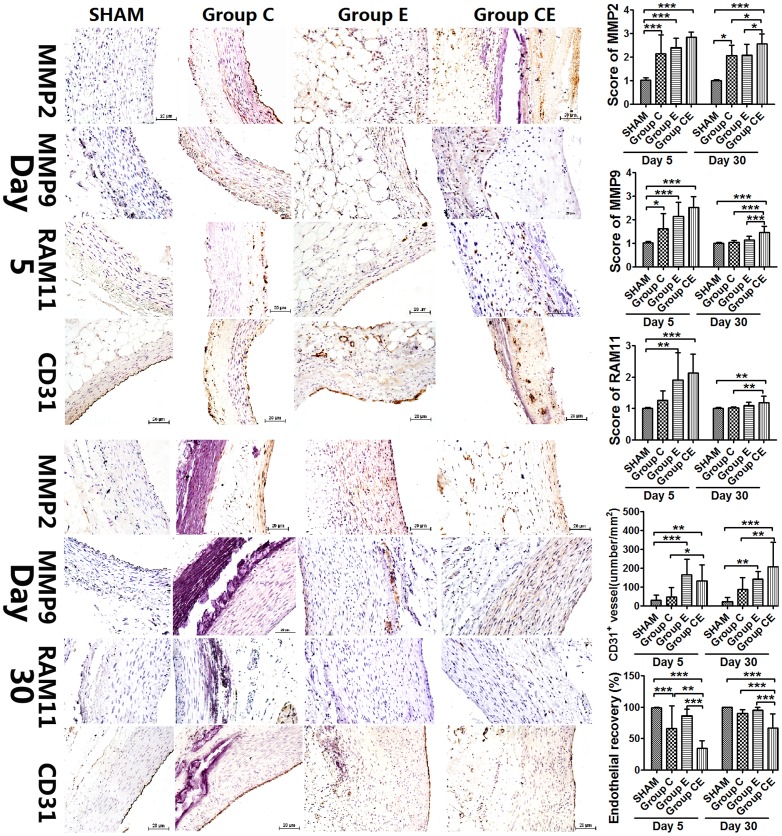
Immunohistochemical changes on day 5 and 30 after surgery, and scores of MMP2, MMP9, RAM11 expression and capillary density as well as endothelial recovery. *P<0.05, **P<0.01, ***P<0.0001. Bar = 20 µm, original magnification ×200.

Compared to the Sham Group, MMP2 and MMP9 expression increased significantly after 5 days in other groups and RAM11 expression in Group E and Group CE was also stronger (*P*<0.01, *P*<0.0001, respectively). Thirty days after surgery, MMP2 still showed a moderate expression, however, MMP9 expression was almost nonexistent and very weak expression of RAM11 was seen in the adventitia in Group C. Both MMP9 and RAM11 showed weak expression in Group E, which was confined to the adventitia of incubated aortic wall, and no expression was found in the media and hyperplastic intima. Adventitial inflammatory cell infiltration, mainly macrophages, was weak in the control groups (Figure 4). Immunostaining in Group CE demonstrated moderate levels of MMP2 and MMP9 protein expression and weak expression of RAM11 throughout the incubated aortic wall. All expressions were still higher in Group CE than the control groups even after 30 days, which might cause the progression of aneurysm gradually in this group. Capillary density was higher in Group E and Group CE compared with Group C and the Sham Group on day 5. CD31^+^ vessels increased significantly in Group CE compared with Group C and the Sham Group after 30 days (*P*<0.01). Sections immunostained with CD31 for endothelial cells showed lower recovery of the endothelium in Group C, Group E and Group CE than the Sham Group, endothelial recovery in Group CE was lowest, which was significantly lower than those in Group C and Group E on day 5. Thirty days later, endothelial recovery in Group CE was lower than other three groups (*P*<0.0001).

### SMC Content Changes

**Figure 5:**
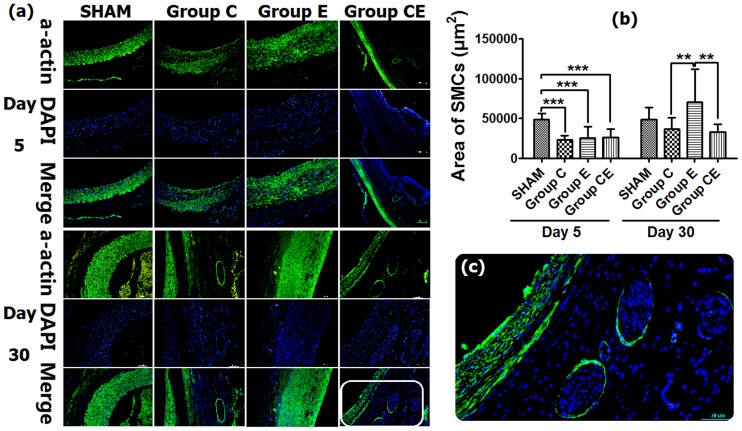
SMC changes viewed with immunofluorescent staining (a) and analysis result of SMC content (b). (**c**) Detailed micrograph of alpha-actin staining in Group CE on day 30. **P<0.01, ***P<0.0001. Original magnification ×200.

Immunofluorescent staining of SMCs indicated that SMC content decreased significantly in all groups except for the Sham Group on day 5 (*P*<0.0001). SMCs were almost always seen in the media and hyperplastic intima of aortic wall on day 30. Thirty days later, SMC content increased significantly in Group E compared to Group C and Group CE (*P*<0.01), SMC content in Group C and Group CE were lower than that in the Sham Group, although the loss of SMCs was not significant (Figure 5). Interestingly, as depicted in figure 5c, the medial layer of Group CE showed almost absent alpha-SMA-positive cells, while these cells were very dense in the hyperplastic endothelial layer. This might be caused by endothelial-to-mesenchymal transition.

## Discussion

The native AAA model induced by elastase perfusion is a standard aneurysm model for experimental research in rodents [3], [22], [23], [24], [25], [26], [27], [28] and rabbits [29], [30], [31]. Anidjar et al. [3] first introduced this method to create an AAA model in rats and suggested that elastase can lead to AAA development through enhanced inflammation response, elastolysis and subsequent destruction of the aortic walls. In this popular model, a catheter is inserted into the femoral artery, the isolated aorta is clamped to block blood circulation and the femoral artery is ligated and subsequently sutured. These procedures make this model more complex and less accessible than models created by adventitial elastolysis that are created by bathing infra-renal aorta segments in elastase solution for 70 min [10] or up to 3 hours [8], [9], [11]. The incubation is obviously lengthy, which might result in high mortality or other failures of the surgical procedure.

A CaCl_2_-induced aneurysm model is another standard aneurysm model in rodents [32], [33], [34], [35] and rabbits [12], [13], [36]. Due to the high affinity of calcium for elastin, calcium deposition within the elastic network of the media can cause the calcium–elastic tissue complex to weaken the vessel wall and develop aneurysm [12]. There is a temporal correlation between inflammatory infiltration and enlargement of aortic aneurysm in vivo [22]. This complex can also serve as the focus of inflammatory, arteriosclerotic reaction of the aortic wall and subsequent aortic aneurysm development [12]. Tanaka et al. developed a novel rat AAA model using a combination of intraluminal elastase infusion and extraluminal CaCl_2_ exposure [15]. We altered the elastase administration by periaortic incubation and simplified this operation dramatically. Periaortic incubation also avoids contact with blood, which is beneficial because elastase inhibition can be reduced by rabbit serum [37]. Moreover, rabbit aneurysms are suitable for performing endovascular aneurysm repair and for investigating the pharmacologic or gene therapeutic effects from drug-eluting stents or stent graft-mediated gene delivery systems. It is quite obvious that the novel rat AAA model induced by Tanaka et al. is too small to perform aneurysm repair. Zhong et al. [17] successfully implanted partially covered polyester stent grafts in rabbit balloon-injured aortas, and our model will be helpful for further endovascular research by stent graft.

In the present study, we have successfully induced a novel rabbit AAA model through a combination of periaortic CaCl_2_ and elastase incubation. Aorta in Group CE achieved sufficient aneursymal dilation, with a high success rate of AAA formation after a 20-minute incubation period. This aneurysm showed more infiltration of macrophages and neovascularization in the aortic wall, more upregulation of MMP2 and MMP9 expression in the media, but less elastin content, endothelial recovery and a lower SMC content. As reported in rat models, the elastase infusion precipitated significant loss of SMCs and endothelial denudation [38]. Balloon injury results in complete denudation of the endothelial cells, which might lead to migration and proliferation of medial SMCs to the intima [39]. Intimal thickening after injury consisted of an endothelial covering and many SMCs. Endothelial recovery was lowest in Group CE on day 5 due to the combined injury of elastase and calcium chloride. Reendothelialization was slower and endothelial covering was incomplete in Group CE on day 30. Additionally, the increased capillary density in Group CE might correlate with an ongoing state of inflammation.

The synergistic effects of CaCl_2_ and elastase stimulates an elastolytic cascade and inflammatory response in the aortic walls [15], and result in the continual progress of aneurysm development, which is quite different from segments solely incubated by elastase or CaCl_2_. In Group E, only 1 of 6 rabbits showed successful AAA on day 5, indicating that our elastase solution of 1 Unit/µL was not effective enough to induce AAA. Also, there was no AAA development in the CaCl_2_-induced rabbit (Group C), although intimal thickening and medial calcification were seen. This result indicated that periaortic incubation of 0.5 mol/L CaCl_2_ for 20 min causes an increase in wall thickness with extensive intimal hyperplasia and calcification, but no formation of aneurysm. Freestone et al. [13] reported the same findings, there was no aneurysmal dilatation of the aorta after periaortic application of 0.5 mol/L CaCl_2_, and rapid aneurysmal dilatation only occurred after periaortic application of CaCl_2_ and thioglycollate in cholesterol-fed rabbits. However, the SMC, elastin and type III collagen content decreased significantly in Group C, indicating that periaortic CaCl_2_ incubation might promote aneurysm formation and progression when combined with elastase incubation. Of note, there was no atherosclerosis in our model, and a cholesterol-enriched diet was important in CaCl_2_-induced AAA formation. We did not perform any pharmacologic manipulation, such as doxycycline, propranolol or simvastatin with this model to demonstrate an impact in aneurysm formation. We will study the influence of pharmacologic intervention on aneurysm formation and development in our novel model in the future.

In conclusion, we present an easy, efficient and reproducible way to create rapid dilation of rabbit aortic arteries to form a model of AAA. This simpler aneurysm model could be valuable for elucidating AAA mechanisms and therapeutic interventions, especially through drug-eluting stents or stent graft-mediated gene delivery system in experimental studies.
